# Clinical outcomes of postoperative radiotherapy with regional nodal irradiation excluding internal mammary lymph nodes in breast cancer: a multi-institutional retrospective analysis

**DOI:** 10.1007/s12282-026-01864-1

**Published:** 2026-05-18

**Authors:** Kanako Nakatsu, Yuka Ono, Michio Yoshimura, Kimiko Hirata, Chikako Yamauchi, Masakazu Ogura, Takahiro Kishi, Kota Fujii, Shuji Ohtsu, Takashi Sakamoto, Kazuhito Ueki, Kengo Ogura, Setsuko Okumura, Itaru Ikeda, Takamasa Mitsuyoshi, Masaki Kokubo, Takashi Mizowaki

**Affiliations:** 1 Department of Radiation Oncology and Image-Applied Therapy, Graduate School of Medicine, Kyoto University, 54 Shogoin-Kawahara-cho, Sakyo-ku, Kyoto, 606-8507 Japan; 2 Department of Radiation Oncology, Kyoto City Hospital, 1-2 Mibu Higashitakada-cho, Nakagyo-ku, Kyoto, 604-8845 Japan; 3 Department of Radiation Oncology, Shiga General Hospital, 5-4-30, Moriyama, Shiga 524-0022 Japan; 4 Department of Radiation Oncology, Kishiwada City Hospital, 1001 Gakuhara-cho, Kishiwada, Osaka 596-8501 Japan; 5 Department of Radiation Oncology, Osaka Red Cross Hospital, 5-30 Fudegasaki-cho, Tennoji-ku, Osaka, 543-8555 Japan; 6 Department of Radiation Oncology, Kurashiki Central Hospital, 1-1-1 Miwa, Kurashiki, Okayama, 710-8602 Japan; 7 Department of Radiation Oncology, Kyoto Katsura Hospital, 17 Yamada-Hirao-cho, Nishikyo-ku, Kyoto, 615-8256 Japan; 8 Department of Radiation Oncology, National Hospital Organization Kyoto Medical Center, 1-1 Fukakusa Mukaihata-cho, Fushimi-ku, Kyoto, 612-0861 Japan; 9 Department of Radiation Oncology, Japanese Red Cross Wakayama Medical Center, 4- 20 Komatsubara-dori, Wakayama, 640-8558 Japan; 10 Department of Radiation Oncology, Hyogo Prefectural Amagasaki General Medical Center, 2-17-77 Higashi-Naniwa-cho, Amagasaki, 660-8550 Hyogo Japan; 11 Department of Radiation Oncology, Kobe City Medical Center General Hospital, 2-1-1 Minatojimaminamimachi, Chuo-ku, Kobe, 650-0047 Hyogo Japan

**Keywords:** Regional nodal irradiation, Internal mammary node region, Breast cancer, Postoperative radiation therapy, Disease-free survival

## Abstract

**Background:**

Postoperative regional nodal irradiation (RNI) is a standard treatment for breast cancer at high risk of regional recurrence; however, the necessity of including the internal mammary node (IMN) region in the radiation field remains unclear. This study aimed to evaluate treatment outcomes in a large cohort of patients who received postoperative radiotherapy with RNI excluding the IMN region.

**Methods:**

This study included patients with breast cancer who underwent surgery followed by RNI without IMN irradiation between 2007 and 2018. The primary endpoint was disease-free survival (DFS), and the secondary endpoints were overall survival (OS), breast cancer-specific mortality (BCM), distant metastasis-free survival (DMFS), recurrence patterns, and treatment-related adverse events.

**Results:**

In total, 799 patients were included. The 5-year DFS, OS, BCM, and DMFS rates were 75.9, 88.3, 9.7, and 77.1%, respectively. Worse outcomes were associated with a higher number of positive lymph nodes and estrogen receptor (ER)-negative disease. Medial/central tumor location and younger age were each significantly associated with poorer outcomes, being associated with worse DFS and DMFS. Bone was the most common recurrence site. ER-negative disease, a higher number of positive lymph nodes, medial/central location, and younger age were significant risk factors for recurrence, particularly distant metastasis. IMN recurrence was rare.

**Conclusions:**

In this cohort, medial/central tumor location, ER-negative disease, and extensive nodal involvement were associated with poorer outcomes, suggesting that these factors may identify patients who can benefit from IMN irradiation. These findings may serve as important reference data when determining the indication for IMN irradiation on an individual patient basis.

**Supplementary Information:**

The online version contains supplementary material available at 10.1007/s12282-026-01864-1.

## Introduction

Breast cancer is the most commonly diagnosed malignancy among women worldwide. According to global estimates from the International Agency for Research on Cancer (Global Cancer Observatory), approximately 2.3 million new cases of breast cancer and 670,000 related deaths were reported globally in 2022, making it the leading cause of cancer incidence and one of the leading causes of cancer-related mortality among women. Despite substantial improvements in early detection and systemic therapies, the global burden of breast cancer continues to increase and is projected to further increase in the coming decades [1].

For early-stage breast cancer, including ductal carcinoma in situ (stage 0) and stage I/II invasive disease, standard local treatment options include mastectomy and breast-conserving therapy (BCT). BCT consists of lumpectomy followed by whole-breast irradiation (WBI) and is recognized as the standard of care. The efficacy of postoperative WBI in stage I and II breast cancer has been demonstrated through multiple randomized controlled trials [2–8], showing that ipsilateral breast tumor recurrence is reduced to approximately one-third and that overall survival (OS) may also be improved [9].

Postoperative regional nodal irradiation (RNI) is also considered for selected patients based on surgical modality and lymph node status. For patients with axillary lymph node involvement undergoing breast-conserving surgery, the addition of RNI is recommended. For patients undergoing total mastectomy, postmastectomy radiation therapy (PMRT), typically including irradiation of the chest wall and regional lymph nodes, is advised for T3 or larger tumors, positive surgical margins, or confirmed nodal metastasis [10–12]. Although irradiation of the supraclavicular region is widely accepted, whether the internal mammary lymph node (IMN) region should be included remains controversial.

Several clinical studies have examined the effectiveness of IMN irradiation (IMNI). Two randomized controlled trials conducted in France and South Korea directly compared clinical outcomes with and without IMNI. The French trial found no significant difference in 10-year OS between groups [13], although limitations included a high proportion of T1–2 tumors, a substantial subset of node-negative patients, and use of outdated two-dimensional planning techniques. Similarly, the South Korean trial did not demonstrate a significant benefit of IMNI in the overall study population [14].

In contrast, observational studies and meta-analyses have supported potential benefits of IMNI. A prospective cohort study in Denmark (DBCG-IMN1) demonstrated improved OS, BCM, and distant metastasis rates in patients who received IMNI [15]. The subsequent DBCG-IMN2 study showed that IMNI continued to reduce 15-year BCM and distant metastasis rates and improved OS, even with modern systemic therapies [16]. A large meta-analysis from China reported an 11% reduction in overall mortality and improved DFS among patients with N1 or N2 disease who received IMNI [17].

Furthermore, the MA.20 [18] and EORTC 22,922/10,925 [19] trials included the IMN region within broader nodal irradiation protocols and showed reductions in recurrence and mortality in their meta-analyses [10]. However, whether these improvements were driven by IMNI or supraclavicular irradiation remains unclear.

This study aimed to evaluate treatment outcomes in patients with breast cancer who received RNI excluding the IMN region using real-world data. In the context of ongoing debate and lack of consensus regarding routine IMNI, these findings may inform future treatment strategies and guide refinements in guidelines for nodal irradiation. To the best of our knowledge, this is the first multi-institutional study to report the clinical outcomes of RNI excluding the IMN region.

## Patients and methods

### Study design

This retrospective cohort study was conducted across 11 institutions affiliated with the Kyoto Radiation Oncology Study Group (KROSG) to evaluate the clinical outcomes of patients with breast cancer who received RNI without IMNI.

Eligible patients had pathologically confirmed breast cancer and underwent definitive surgical resection, either mastectomy or breast-conserving surgery, with or without reconstruction. Patients were required to have received chest wall or WBI combined with RNI *using three-dimensional conformal radiotherapy (3D-CRT)* between January 1, 2007, and December 31, 2018, and to have completed the planned radiotherapy (RT) course. The exclusion criteria were IMNI as part of initial treatment, distant metastasis at diagnosis, prior chest wall irradiation, or a follow-up period of < 3 months from RT initiation. *Patients with clinically evident IMN metastasis were also excluded from this cohort. IMN involvement was assessed on the basis of imaging findings obtained before postoperative radiotherapy. Because this was a multi-institutional retrospective study*,* the specific diagnostic modalities used to evaluate IMN involvement varied across institutions and could not be collected comprehensively. However*,* at Kyoto University Hospital*,* for example*,* breast MRI was performed in 85 of 97 patients (87.6%) and FDG-PET/CT in 42 patients (43.3%; with some overlap) during the initial staging workup.*

The primary endpoint was DFS, defined as the interval from the start of RT to recurrence, distant metastasis, or death from any cause. The secondary endpoints were as follows: OS, defined as survival irrespective of cause of death; BCM, defined as death attributable to breast cancer; and distant metastasis-free survival (DMFS), defined as survival without occurrence of distant metastasis. The first site of recurrence was classified into three categories: local recurrence in the ipsilateral breast or chest wall; regional nodal recurrence in the ipsilateral axillary, supraclavicular, or IMN; and distant recurrence in non-irradiated skin or subcutaneous tissue, bone, lung, liver, or other organs, including contralateral lymph nodes. Recurrences in marginal skin or subcutaneous areas potentially receiving insufficient radiation dose were also classified as distant recurrence. Radiation-related adverse events were assessed using the Common Terminology Criteria for Adverse Events version 5.0. Clinical, pathological, treatment, and follow-up data were retrospectively collected from institutional databases, with June 30, 2022, as the data cutoff date.

### Statistical analyses

DFS, OS, BCM, and DMFS were estimated using the Kaplan–Meier method. *Univariable* analyses were performed using the log-rank test or *univariable* Cox proportional hazards model, as appropriate. *Multivariable analyses were performed using Cox proportional hazards regression models to evaluate the independent prognostic impact of clinical and pathological variables. Age at first visit*,* laterality (left vs. right)*,* use of taxane-based chemotherapy*,* estrogen receptor (ER) status*,* primary tumor location*,* and the number of metastatic lymph nodes were included based on clinical relevance and previously reported prognostic factors*,* while limiting the number of variables to avoid model overfitting. T category and histological grade were excluded due to substantial missing data*,* and triple-negative subtype was not included because ER status was already included in the model.*

For BCM, where non-breast cancer-related death was considered a competing event, cumulative incidence functions were estimated using the Fine–Gray subdistribution hazard model, which was also used for the *multivariable* analysis of BCM to account for competing risks.

Logistic regression analysis was performed to identify factors associated with different initial recurrence patterns (locoregional, IMN, and distant). Explanatory variables included age at first visit, laterality (left vs. right), use of taxane-based chemotherapy, *ER* status, primary tumor location, and the number of metastatic lymph nodes. The association between primary tumor location and IMN recurrence was examined using Fisher’s exact test.

Statistical significance was set at a two-sided *p* < 0.05. Statistical analyses were performed using EZR version 1.68 (Saitama Medical Center, Jichi Medical University, Saitama, Japan), a graphical interface for R [20].

## Results

### Patient population

From January 2007 to December 2018, 799 eligible patients were identified across 11 KROSG-affiliated institutions. The number of enrolled patients per institution is shown in Online Resource 1.

The median age at the start of RT was 59 (range, 24–93) years. Most patients (81.5%) received 50 Gy in 25 fractions (fr), whereas 17.8% received 60 Gy in 30 fr (50 Gy/25 fr + 10 Gy/5 fr boost to the tumor bed, positive surgical margins, and supraclavicular and axillary lymph node regions). Tumor location was lateral in 51.2% of the patients and medial/central in 46.1% of the patients. *Systemic therapies*,* including chemotherapy*,* endocrine therapy*,* and anti-human epidermal growth factor receptor 2 (HER2) therapy*,* were administered in accordance with contemporary treatment guidelines for patients with appropriate indications*,* unless medically contraindicated or declined by the patient. In this cohort*,* 87.5% of patients received neoadjuvant or adjuvant chemotherapy*,* including taxane-based regimens in 75.2% of those treated.* Patients’ baseline characteristics are summarized in Table 1.

**Table 1:** Baseline patient and tumor characteristics

Age, median (range), y	*N*	%
	54 (24–93)	
*Laterality*		
Right	414	51.8
Left	385	48.2
*Tumor location*		
Lateral	409	51.2
Medial/central	368	46.0
Unknown	22	2.8
*Histological type*		
Ductal	714	89.4
Lobular	52	6.5
Others or unknown	33	4.1
*cT category*		
T0	5	0.6
is	6	0.8
T1	144	18.0
T2	394	49.3
T3	83	10.4
T4	137	17.1
Unknown	30	3.8
*cN category*		
N0	212	26.5
N1	415	51.9
N2	79	9.9
N3	64	8.0
Unknown	29	3.7
*pT or ypT category*		
T0	72	9.0
Tis	18	2.3
T1	218	27.3
T2	307	38.4
T3	97	12.1
T4	78	9.8
Unknown	9	1.1
*pN or ypN category*		
N0	158	19.8
N1	299	37.4
N2	218	27.3
N3	117	14.6
Unknown	7	0.9
No. of dissected nodes, median (range)	15 (0–61)	
No. of positive nodes, median (range)	3 (0–60)	
0	157	19.6
1–3	262	32.8
≥ 4	370	46.3
Unknown	10	1.3
*Resection margin*		
Negative	692	86.6
Positive	84	10.5
Unknown	23	2.9
*Histological grade*		
1	109	13.6
2	333	41.7
3	170	21.3
Unknown	187	23.4
*ER*		
Negative	195	24.4
Positive	599	75.0
Unknown	5	0.6
*HER2 status*		
0	298	37.3
1	248	31.0
2	132	16.5
3	115	14.4
Unknown	6	0.8
*IHC subtype*		
ER–/HER2–	114	14.3
ER–/HER2+	81	10.1
ER+/HER2–	433	54.2
ER+/HER2+	166	20.8
Unknown	5	0.6
*Chemotherapy*		
Taxane-containing regimen	601	75.2
Non-taxane-containing regimen	84	10.5
Unknown regimen	2	0.3
No	100	12.5
Unknown	12	1.5
*Surgery type*		
Breast-conserving surgery	249	31.2
Mastectomy	549	68.7
Others	1	0.1
*Location and lymph nodes*		
Lateral, no positive node	79	9.9
Medial/central, no positive node	78	9.8
Lateral, 1–3 positive nodes	119	14.9
Medial/central, 1–3 positive nodes	135	16.9
Lateral, ≥ 4 positive nodes	208	26.0
Medial/central, ≥ 4 positive nodes	153	19.1
Unknown	27	3.4
Total irradiated dose, median (range)	50 (50–66) Gy	

*ER* estrogen receptor; *HER2* human epidermal growth factor receptor 2

## Outcomes

**Fig. 1:**
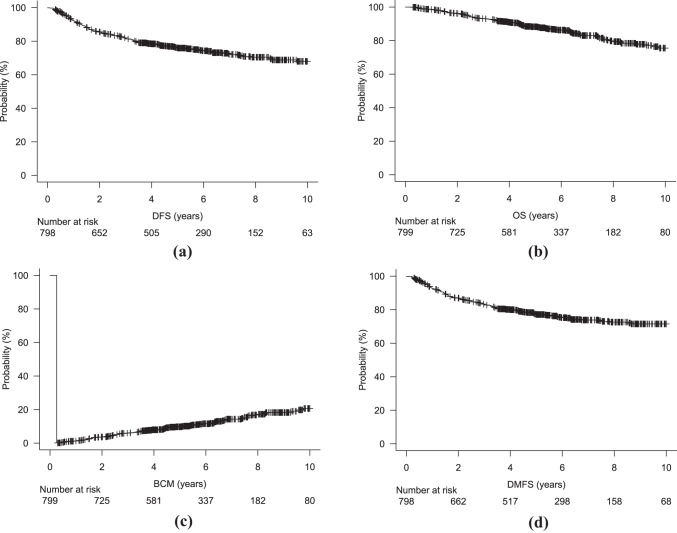
Kaplan–Meier survival curves: (**a**) Disease-free survival, **b** overall survival, **c** breast cancer-specific mortality, and **d** distant metastasis-free survival

The median follow-up period for OS was 5.4 (range, 0.26–14.9) years. The 5-year DFS rate was 75.9% (95% confidence interval [CI], 72.6–78.8%; Fig. 1a). The 5-year OS, BCM, and DMFS rates were 88.3% (95% CI, 85.6–90.4%), 9.7% (95% CI, 7.7–12.2%), and 77.1% (95% CI, 73.9–80.0%), respectively (Fig. 1b and d).

*Univariable* analysis showed that increasing numbers of positive lymph nodes and ER-negative disease were associated with poorer DFS, OS, BCM, and DMFS (Table 2). *Multivariable* analyses confirmed these findings (Table 3) and additionally identified medial/central tumor location and younger age at diagnosis as adverse prognostic factors for DFS and DMFS.

**Table 2:** *Univariable* analysis of prognostic factors for DFS, OS, BCM, and DMFS

		DFS		OS		BCM		DMFS	
		HR (95% CI)	*P*-value	HR (95% CI)	*P*-value	HR (95% CI)	*P*-value	HR (95% CI)	*P*-value
Age	Continuous	0.99 (0.98–1.00)	0.14	1.02 (1.00–1.03)	0.05	1.00 (0.99–1.02)	0.63	0.99 (0.98–1.01)	0.27
Tumor location	Lateral vs. medial/central	1.32 (0.99–1.75)	0.06	1.25 (0.86–1.80)	0.24	1.20 (0.80–1.80)	0.39	1.32 (0.99–1.77)	0.06
Laterality	Right vs. left	0.88 (0.67–1.16)	0.14	0.95 (0.67–1.36)	0.79	0.91 (0.62–1.36)	0.66	0.82 (0.62–1.10)	0.18
ER	Positive vs. negative	1.80 (1.34–2.42)	**< 0.001**	1.97 (1.35–2.88)	**< 0.001**	2.25 (1.50–3.39)	**< 0.001**	1.89 (1.39–2.56)	**< 0.001**
Taxane regimen	No vs. yes	1.14 (0.82–1.60)	0.44	0.99 (0.65–1.51)	0.95	1.26 (0.76–2.09)	0.36	1.14 (0.80–1.61)	0.45
No. of positive LNs	0 vs. 1–3 vs. ≥4	1.55 (1.27–1.90)	**< 0.001**	1.74 (1.32–2.31)	**< 0.001**	1.87 (1.36–2.58)	**< 0.001**	1.61 (1.30–1.98)	**< 0.001**

*DFS* disease-free survival, *OS* overall survival, *BCM* breast cancer-specific mortality, *DMFS* distant metastasis-free survival, *HR* hazard ratio, *CI* confidence interval, *ER* estrogen receptor, *LN* lymph node

**Table 3:** *Multivariable* analysis of prognostic factors for DFS, OS, BCM, and DMFS

		DFS		OS		BCM		DMFS	
		HR (95% CI)	*P*-value	HR (95% CI)	*P*-value	HR (95% CI)	*P*-value	HR (95% CI)	*P*-value
Age	Continuous	0.99 (0.97–1.00)	**0.023**	1.01 (0.99–1.03)	0.26	1.00 (0.98–1.02)	0.86	0.99 (0.97–1.00)	**0.031**
Tumor location	Lateral vs. medial/central	1.48 (1.11–1.98)	**0.008**	1.43 (0.98–2.10)	0.06	1.42 (0.93–2.17)	0.10	1.51 (1.12–2.04)	**0.007**
Laterality	Right vs. left	0.86 (0.65–1.15)	0.32	0.93 (0.63–1.36)	0.70	0.86 (0.56–1.32)	0.49	0.83 (0.61–1.12)	0.21
ER	Positive vs. negative	2.53 (1.81–3.52)	**< 0.001**	2.53 (1.65–3.88)	**< 0.001**	3.04 (1.91–4.83)	**< 0.001**	2.67 (1.90–3.74)	**< 0.001**
Taxane regimen	No vs. yes	0.81 (0.56–1.18)	0.28	0.88 (0.54–1.42)	0.59	1.04 (0.58–1.85)	0.91	0.81 (0.55–1.19)	0.29
No. of positive LNs	0 vs. 1–3 vs. ≥4	1.77 (1.44–2.18)	**< 0.001**	1.94 (1.46–2.59)	**< 0.001**	2.14 (1.55–2.97)	**< 0.001**	1.87 (1.50–2.32)	**< 0.001**

*DFS* disease-free survival, *OS* overall survival, *BCM* breast cancer-specific mortality, *DMFS* distant metastasis-free survival, *HR* hazard ratio, *CI* confidence interval, *ER* estrogen receptor, *LN* lymph node

In the Fine–Gray competing risk model for BCM, significant predictors of worse prognosis included increasing numbers of metastatic lymph nodes (hazard ratio [HR], 2.11; 95% CI, 1.50–2.98; *p* < 0.0001) and ER-negative disease (HR, 3.08; 95% CI, 1.85–5.11; *p* < 0.0001).

Initial recurrence sites, including overlapping events, are shown in Table 4. Bone was the most common site of recurrence, followed by the lungs and liver. The probabilities of initial recurrence were 3.1% for ipsilateral breast and chest wall, 4.9% for regional lymph nodes including 2.1% for IMN, and 22.7% for distant metastases.

**Table 4:** Initial sites of recurrence, including overlapping cases

Site	*N*	%
Bone	103	12.9
Lung	66	8.3
Liver	54	6.8
Brain	34	4.3
Breast or chest wall	25	3.1
Regional lymph node(s)	39	4.9
Axillary and/or supraclavicular lymph node(s)	22	2.8
IMN	17	2.1
Skin (outside the irradiation field)	8	1.0
Skin (at the edge of the irradiation field)	4	0.5
Others^*^	47	5.9

*Pericardium, distant lymph nodes (abdominal, cervical, contralateral axillary, mediastinal), pancreas, adrenal glands, retroperitoneum, stomach, intramuscular, and bone marrow

IMN, internal mammary node

Logistic regression results according to recurrence patterns are shown in Table 5. ER-negative disease was associated with increased risk of ipsilateral breast/chest wall recurrence (odds ratio [OR], 3.77; 95% CI, 1.59–8.93; *p* = 0.003). For regional lymph node recurrence, ER-negative disease (OR, 6.29; 95% CI, 2.51–15.8; *p* < 0.001) and ≥ 4 metastatic lymph nodes (OR, 13.4; 95% CI, 1.69–106; *p* = 0.014) were adverse factors. For distant metastases, younger age at diagnosis (OR, 0.98; 95% CI, 0.97–1.00; *p* = 0.009), ER-negative disease (OR, 2.51; 95% CI, 1.65–3.82; *p* < 0.001), increased number of metastatic lymph nodes (OR, 1.08; 95% CI, 1.05–1.11; *p* < 0.001), and medial/central tumor location (OR, 1.84; 95% CI, 1.28–2.64; *p* < 0.001) were significant predictors.

**Table 5:** Logistic regression analysis of risk factors associated with recurrence at each site

		Ipsilateral breast or chest wall		Regional lymph nodes		Distant metastasis	
		OR (95% CI)	*P*-value	OR (95% CI)	*P*-value	OR (95% CI)	*P*-value
Age	Continuous	0.98 (0.94–1.01)	0.18	1.02 (0.98–1.06)	0.31	0.98 (0.97–1.00)	**0.009**
Tumor location	Lateral vs. medial/central	0.79 (0.34–1.82)	0.57	1.01 (0.42–2.45)	0.99	1.84 (1.28–2.64)	**< 0.001**
Laterality	Right vs. left	1.83 (0.80–4.20)	0.16	1.57 (0.65–3.80)	0.31	0.72 (0.51–1.03)	0.07
ER	Positive vs. negative	3.77 (1.59–8.93)	**0.003**	6.29 (2.51–15.8)	**< 0.001**	2.51 (1.65–3.82)	**< 0.001**
Taxane regimen	No vs. yes	1.16 (0.38–3.61)	0.79	1.71 (0.45–6.49)	0.43	0.77 (0.49–1.20)	0.25
No. of positive LNs	0 vs. 1–3	2.17 (0.60–7.82)	0.24	7.20 (0.83–62.1)	0.07	2.11 (1.16–3.84)	**0.015**
	༐ vs. ≥4	2.41 (0.72–8.03)	0.15	13.4 (1.69–106)	**0.014**	4.30 (2.43–7.59)	**< 0.001**

*OR* Odds ratio, *CI* confidence interval, *ER* estrogen receptor, *LN* lymph node

*Multivariable* analysis of IMN recurrence was not feasible due to the small number of events. Fisher’s exact test did not demonstrate a significant association between IMN recurrence and tumor location (*p* = 0.127). The characteristics of *17* patients who developed IMN recurrence are summarized in Online Resource 2.

Radiation dermatitis was the most frequent radiation-related adverse event, occurring as grade 2 in 102 (13%) patients and grade 3 in 8 (1.0%) patients. Other adverse events included lymphedema, radiation pneumonitis, necrosis of the reconstructed breast, one case of grade 5 hepatic failure of uncertain etiology (possibly related to oral tegafur–uracil), and one case of grade 3 heart failure occurring 8.5 years after RT. Details are summarized in Table 6.

**Table 6:** Treatment-related adverse events

Event	Grade	*N*	%
Radiation dermatitis	2	102	12.8
	3	8	1.0
	≥ 4	0	0
Radiation pneumonitis	2	7	0.9
	3	1	0.1
	≥ 4	0	0
Lymphedema	≥ 2	48	6.0
Necrosis of the reconstructed breast	NA	1	0.1
Heart failure	3	1	0.1
Hepatic failure	5	1	0.1

*NA* not available

## Discussion

Postoperative RNI, based on the tumor-node-metastasis classification, has been established as a standard treatment for breast cancer. According to the American Society for Radiation Oncology–American Society of Clinical Oncology–Society of Surgical Oncology Joint Clinical Practice Guidelines, inclusion of the IMN region within the PMRT field is recommended for patients with clinically or radiographically detected IMN nodes and those with *centrally* or medially located breast tumors, particularly when axillary lymph nodes are positive [21]. Consequently, clinical data on outcomes of PMRT with RNI excluding the IMN region are limited. However, in Japan, PMRT excluding the IMN region has historically been performed in patients diagnosed with IMN-negative based on detailed preoperative imaging assessments, resulting in the accumulation of a substantial number of such cases.

Kim et al. [14] reported that, in the overall study population of the KROG 0806 trial, there were no significant differences between the non-IMNI and IMNI groups in 7-year DFS (81.9% vs. 85.3%), OS (88.2% vs. 89.4%), or BCM (10.8% vs. 8.4%). However, ad hoc subgroup analyses demonstrated that IMNI significantly improved 7-year DFS and BCM in patients with medially or centrally located tumors, without increasing the incidence of adverse events. In the DBCG-IMN1 trial, Thorsen et al. [15] reported improved long-term outcomes with IMNI. Specifically, the IMNI group showed higher OS and lower BCM at both 8 years (OS, 72.2% vs. 75.9%; BCM, 23.4% vs. 20.9%) and 15 years (OS, 55.4% vs. 60.1%; BCM, 33.9% vs. 31.7%) compared with the non-IMNI group. In the DBCG-IMN2 trial, Nielsen et al. [16] demonstrated that IMNI was associated with improved long-term outcomes even in the era of modern systemic therapies, including taxane-based chemotherapy, anti-HER2 therapy, and aromatase inhibitors. At 15 years, the IMNI group showed higher OS (60.8% vs. 65.0%), lower BCM (23.6% vs. 21.4%), and lower distant recurrence rates (26.9% vs. 25.1%) compared with the non-IMNI group. A meta-analysis by Jia et al. [17] analyzed 12,705 patients from 12 studies and reported that IMNI reduced mortality and improved DFS in patients with N1 and N2 diseases. A recent Japanese study reported treatment outcomes of postoperative RT excluding the IMN region, demonstrating that IMN failure was rare and that hormone receptor–negative disease was an independent risk factor for IMN recurrence [22]; thus, the present multi-institutional study of 799 patients across 11 institutions provides complementary real-world insights into clinical outcomes without IMNI.

Compared with previous studies, particularly the KROG 0806 [14] and DBCG-IMN1,2 [15, 16] trials, our DFS, OS, and BCM results were generally consistent with non-IMNI groups, although our cohort included some patients with N0 disease. Notably, a high proportion of patients with nodal metastases had ≥ 4 positive lymph nodes, representing a high-risk population.

Recurrence patterns showed that IMN recurrences were low despite the omission of IMNI. However, this finding does not justify excluding IMNI, as sentinel lymph node biopsies suggest occult tumor cells may exist in the IMN region even without clinically apparent recurrence [23], potentially contributing to distant metastasis through systemic dissemination [14–16].

*Multivariable* analysis demonstrated that medial/central tumors were associated with poorer DFS, DMFS, and distant metastatic recurrence. As shown in Online Resource 3, even among patients with a higher number of metastatic lymph nodes, lateral tumors were associated with better DFS. In the KROG 0806 trial [14], no significant difference in prognosis was observed in the overall study population between patients with and without IMNI; nevertheless, subgroup analyses demonstrated lower recurrence rates in the irradiated group among medial/central tumors. In contrast, both DBCG-IMN1 and DBCG-IMN2 trials showed improved outcomes with the addition of IMNI in all node-positive patients [15, 16]. In the DBCG-IMN1 trial, subgroup analyses suggested that patients with lateral tumors and one to three positive lymph nodes seemed to have a lower absolute benefit from IMNI. In the DBCG-IMN2 trial, combined subgroup analyses of tumor location and number of positive LN suggested limited benefit from IMNI in patients with medial/central tumors and ≥ 4 positive lymph nodes; accordingly, the authors concluded that no subgroup was identified in which IMNI could be safely omitted. Taken together, although definitive conclusions cannot be drawn regarding tumor location and IMN recurrence, differences in lymphatic flow patterns between medial/central and lateral tumors [24] may underlie the observed prognostic differences, supporting potential benefit of IMNI in medial/central tumors. Our findings are consistent with this concept.

*Notably*,* in the present study*,* ER-negative disease consistently emerged as an unfavorable prognostic factor across multiple clinical endpoints*,* including DFS*,* OS*,* BCM*,* and DMFS. Current clinical guidelines generally recommend considering IMNI*,* based primarily on anatomical risk factors such as tumor location and axillary lymph node status. Importantly*,* previous randomized trials such as MA.20 and KROG 08 − 06 have also suggested that the benefit of regional nodal irradiation may be more pronounced in patients with hormone receptor–negative breast cancer. Taken together*,* these findings suggest that biological factors*,* in addition to conventional anatomical criteria*,* may play an important role in refining patient selection for IMNI.*

Several factors limit routine IMNI in Japan: (1) the necessity of treating the IMN region is questioned when detailed pre-treatment imaging, such as contrast-enhanced magnetic resonance imaging (MRI) or fluorodeoxyglucose positron emission tomography/computed tomography, does not reveal evidence of metastasis; (2) clinically evident IMN recurrence is relatively rare; (3) treatment of IMN recurrence is generally manageable; and (4) IMNI increases cardiac and pulmonary radiation doses, which increases risk of adverse events. Wang et al. reported that among patients without evidence of IMN metastasis on contrast-enhanced MRI, sentinel lymph node biopsy revealed metastasis in 16.9% of cases, suggesting a potential discrepancy between imaging and nodal involvement [25]. The absence of clinically evident IMN metastasis does not rule out the presence of tumor cells in the IMN region; thus, IMNI may contribute to improved disease control and not just prevention of regional recurrence. Advancements in treatment planning (e.g., intensity-modulated radiation therapy and volumetric modulated arc therapy) and delivery methods (e.g., deep inspiration breath hold) have significantly reduced radiation exposure to surrounding organs [26–29], enabling safer delivery of IMNI.

This study has *several* limitations. The retrospective nature of this study introduces potential selection bias, as follow-up is often conducted outside radiation oncology departments, potentially over-representing patients with recurrence and those with poorer prognoses. Systemic therapies and RT for breast cancer have advanced since 2018, with a wider range of medications now available (e.g., cyclin-dependent kinase 4/6 and poly (ADP-ribose) polymerase inhibitors) and safer delivery methods for RT. Anti-HER2 therapy markedly improves the pathological complete response (pCR) rate in HER2-positive breast cancer [30–32]. In the KEYNOTE-522 trial [33], the addition of immune checkpoint inhibitors to chemotherapy was associated with an increased pCR rate in triple-negative breast cancer, whereas the NSABP B-51/RTOG 1304 trial reported that patients with initially node-positive N1 breast cancer who achieved ypN0 after neoadjuvant chemotherapy showed no significant benefit from RNI in terms of invasive recurrence-free interval [34]; hence, the utility of IMNI may be limited in patients who achieve pCR. These advances in therapeutic strategies can potentially influence the current indications for IMNI. *In addition*,* incidental radiation dose to the IMN region cannot be completely excluded. In the DBCG-IMN2 study* [16], *in which treatment allocation was determined according to tumor laterality*,* it was reported that more than 55.8% of the IMN volume was covered by the prescribed dose in 25% of patients in the left-sided treatment group. Furthermore*,* incidental dose to the IMN region has been suggested to be higher in patients with medial/central tumors than in those with lateral tumors. Therefore*,* it cannot be excluded that these factors may have influenced the observed IMN recurrence rate*,* as well as the subgroup comparisons between medial/central and lateral tumors in the present study.*

In this study, we reported the treatment outcomes of Japanese patients with breast cancer who received RNI without IMNI. Although IMNI indications remain controversial, several prospective studies have suggested its potential benefits, particularly for patients with medial/central tumors and extensive axillary lymph node involvement; notably, these same clinicopathological features were associated with significantly worse outcomes in our cohort treated without IMNI. These patients may be appropriate candidates for IMNI, and further studies are warranted to better identify patient selection criteria that predict the benefits of IMNI.

## Supplementary Information

Below is the link to the electronic supplementary material.

Supplementary Material 1
