# Supplementary material for: Clinical outcomes of postoperative radiotherapy with regional nodal irradiation excluding internal mammary lymph nodes in breast cancer: a multi-institutional retrospective analysis
Source: Breast Cancer. 2026 May 18;33(4):876–86. doi: 10.1007/s12282-026-01864-1 (PMC13283185; doi:10.1007/s12282-026-01864-1)
Supplement: Supplementary file 1 — Supplementary Material 1 [file 12282_2026_1864_MOESM1_ESM.pdf]

ESM1

Institution	*Number of cases*
Kyoto University	97
Kishiwada City Hospital	139
Shiga General Hospital	97
Osaka Red Cross Hospital	95
Kurashiki Central Hospital	88
Kyoto City Hospital	61
Kyoto Katsura Hospital	54
National Hospital Organization Kyoto Medical Center	52
Japanese Red Cross Wakayama Medical Center	48
Amagasaki General Medical Center	45
Kobe City Medical Center General Hospital	23
Total	799

ESM2

IMN recurrence	Lateral	Medial/central	Unknown	Total
No	404	357	21	782
Yes	5	11	1	17
Total	409	368	22	799

	N	%
Age, median (range), y	50 (34–78)	
Laterality		
Right	7	41.2
Left	10	58.8
cT category		
T0	1	5.9
T1	1	5.9
T2	8	47.1
T3	4	23.5
T4	3	17.6
cN category		
N0	2	11.8
N1	6	35.3

N2	4	23.5
N3	5	29.4
No. of positive nodes, median (range)	5 (1–42)	
1–3	7	41.2
≥4	10	58.8
ER		
Negative	4	23.5
Positive	13	76.5
HER2 status		
0	5	29.4
1	8	47.1
2	2	11.8
3	2	11.8
Taxane regimen		
No	3	17.6
Yes	14	82.4
Pattern of first recurrence		
IMN only	11	64.7

IMN + regional lymph nodes	4	23.5
IMN + distant metastasis	2	11.8
Time to IMN recurrence (y), median (range)	3.0 (0.3-8.6)	

ESM3

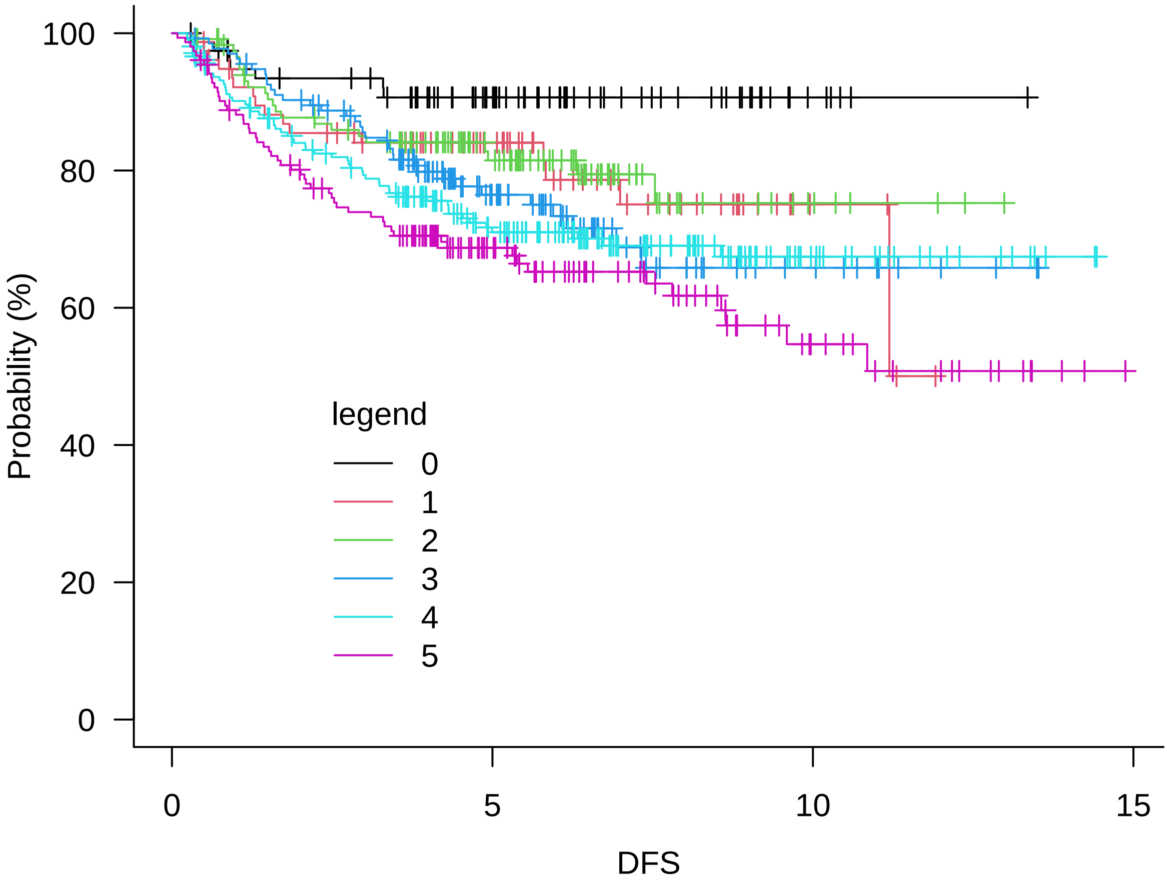

Number at risk

	0	5	10	15
0	79	46	5	0
1	78	41	4	0
2	119	62	6	0
3	135	60	10	0
4	208	102	20	0
5	153	65	17	0

ESM1

The number of enrolled patients per institution.

ESM2

Characteristics of patients with internal mammary node (IMN) recurrence (n=17).

ESM3

Disease-free survival (DFS) stratified by the number of positive lymph nodes and tumor location. Patients were categorized into six groups according to nodal status (0, 1–3, or ≥4 positive lymph nodes) and tumor location (lateral vs mediocentral).

Legend	*Number of positive lymph nodes*	*Tumor location*
0	0	Lateral
1	0	Mediocentral
2	1-3	Lateral
3	1-3	Mediocentral
4	≥4	Lateral
5	≥4	Mediocentral

Article title:

Clinical Outcomes of Postoperative Radiotherapy with Regional Nodal Irradiation Excluding Internal Mammary Lymph Nodes in Breast Cancer: A Multi-Institutional Retrospective Analysis

Journal name: Breast Cancer

Author names:

Kanako Nakatsu, Yuka Ono, Michio Yoshimura, Kimiko Hirata, Chikako Yamauchi, Masakazu Ogura, Takahiro Kishi, Kota Fujii, Shuji Ohtsu, Takashi Sakamoto, Kazuhito Ueki, Kengo Ogura, Setsuko Okumura, Itaru Ikeda, Takamasa Mitsuyoshi, Masaki Kokubo, Takashi Mizowaki

Corresponding author:

Yuka Ono

Department of Radiation Oncology and Image-Applied Therapy, Graduate School of Medicine, Kyoto University, 54 Shogoin-Kawahara-cho, Sakyo-ku, 606-8507, Kyoto, Japan.

yukat@kuhp.kyoto-u.ac.jp
